# Methyl-specific NMR of therapeutic antibodies: cost-effective isotopic labeling strategies in CHO cells for high-resolution structural characterization

**DOI:** 10.1007/s10858-026-00492-3

**Published:** 2026-04-18

**Authors:** Rida Awad, Arthur Giraud, Béatrice Vibert, Séverine Clavier, Hélène Le Borgne, Laetitia Maçon, Anaïs Muhr-Naninck, Stéphanie Seguin-Huet, Benoit Mothes, Pierre Gans, Oriane Frances, Jérôme Boisbouvier, Elodie Crublet

**Affiliations:** 1 NMR-Bio, 225 Route de Bivan, L’Albenc, 38470 France; 2 Sanofi Research & Development, Vitry-sur-Seine, 94403 France; 3 Univ. Grenoble Alpes, CNRS, CEA, Institut de Biologie Structurale (IBS), 71 Avenue des Martyrs, Grenoble, F-38044 France

**Keywords:** Monoclonal antibodies, LAMP1, CHO cells, Isotopic labeling, Higher-order structure (HOS), Methyl NMR

## Abstract

Monoclonal antibodies (mAbs) are essential therapeutic agents whose efficacy depends critically on the integrity of their higher-order structure (HOS). Subtle perturbations in HOS, arising from post-translational modifications, chemical degradation or formulation conditions, can impair antigen binding or induce immunogenicity. Nuclear Magnetic Resonance (NMR) spectroscopy, and particularly methyl NMR, offers a powerful means to probe the structure and dynamics of large proteins, such as antibodies, at atomic scale resolution. While 2D ^1^H-^13^C methyl correlation spectra at natural abundance provide valuable structural fingerprints, their limited resolution and sensitivity hamper sophisticated NMR studies. Isotopic labeling of methyl groups with ^13^CH₃, combined with tailored deuteration, greatly enhances spectral quality. However, such labeling strategies remain underdeveloped in mammalian systems like CHO cells, which are the standard hosts for therapeutic antibody production. Here, we report an optimized labeling strategy enabling selective incorporation of the ¹³CH₃-labeled methyl groups from the six methyl-bearing amino acids into antibodies expressed in CHO cells. This includes enzymatic synthesis of regio- and stereoselectively labeled isoleucine and valine with partial deuteration and an optimized protocol for their incorporation into CHO-produced proteins. When applied to an anti-LAMP1 therapeutic antibody, this strategy yields highly resolved methyl NMR spectra, enabling comprehensive HOS assessment. It allows the transfer of previously assigned Fab and Fc methyl resonances, resulting in approximately 84% of methyl signals being assigned in the intact mAb. This work establishes a robust framework for NMR-based structural analysis of glycosylated antibodies directly from mammalian expression systems.

**Supplementary Information:**

The online version contains supplementary material available at 10.1007/s10858-026-00492-3.

## Introduction

Monoclonal antibodies (mAbs) are central players in the adaptive immune system and have become indispensable tools across biomedical research, diagnostics and therapeutics. Their success in treating numerous pathologies, including cancer, autoimmune and infectious diseases, is underpinned by their high specificity and affinity for a wide range of antigens (Kaplon et al. 2023). Their functional properties are deeply rooted in their higher-order structure (HOS) which refers to the three-dimensional organization of their secondary, tertiary and quaternary elements. This structural hierarchy governs the geometry and accessibility of antigen-binding regions, modulates effector functions via the Fc domain and dictates molecular stability. Even minor perturbations in HOS, whether due to post-translational modifications (PTM), chemical degradation (e.g., oxidation or deamidation) or formulation conditions (for example: pH, ionic strength or excipients), can lead to measurable effects on antigen recognition or immune activation. Consequently, there is a critical need to further develop and expand high-resolution analytical methods for quality control to ensure comprehensive characterization of therapeutic antibody batches, in line with regulatory expectations from authorities such as the FDA and EMA.

Despite this imperative, the analytical methods historically employed in the biopharmaceutical industry, such as differential scanning calorimetry (DSC), circular dichroism (CD), intrinsic fluorescence or Raman spectroscopy (Li et al. 2011, 2024; Hauptmann et al. 2023; Budyak et al. 2024), often lack the resolution to detect subtle structural changes that may nonetheless impact biological function. In recent years, Nuclear Magnetic Resonance (NMR) spectroscopy has emerged as a valuable complement to these techniques, offering distinct advantages over other high-resolution methods such as crystallography or cryo-EM for quality control purposes. NMR provides atomic-level information about protein structure and dynamics in solution, under near-physiological conditions and without the need for crystallization or cryogenic preparation. While cryo-EM excels at providing high-resolution static structures, NMR uniquely captures dynamic conformational ensembles and temporal fluctuations that are directly relevant to therapeutic antibody stability and function. Notably, NMR is capable of detecting both global conformational rearrangements and local microenvironmental changes within complex protein systems under conditions that closely mimic those encountered during manufacturing, storage and administration.

Recent studies have demonstrated the value of natural abundance 2D ^1^H-^13^C methyl NMR in biopharmaceutical contexts for detecting conformational variants, assessing thermal stability, and monitoring antigen binding in mAbs. For instance, pioneering efforts by Arbogast et al. (2015) and Brinson et al. (2019) have shown that well-resolved methyl NMR spectra can serve as “fingerprints” of mAb structural integrity, offering a comprehensive readout of the molecule’s conformational state and stability. More recently, structural alterations induced by methionine oxidation in stressed mAb batches were directly monitored by 2D NMR (Cerofolini et al. 2023; Vibert et al. 2026). In addition to these native-state analyses, PTMs can also be probed under denaturing conditions using ^1^H-^13^C HSQC NMR, where denaturation with deuterated urea simplifies spectra and reveals modifications as additional or shifted signals (Hinterholzer et al. 2019, 2020, 2022; Moises et al. 2023), providing a complementary tool to assess antibody integrity. While natural abundance methyl spectra already provide highly informative fingerprints, their utility in quality control ultimately depends on the ability to assign individual resonances. Signal assignment is essential to establish a robust reference framework, enabling unambiguous monitoring of subsequent natural abundance spectra from production batches. Moreover, such assignments for antibodies expressed in CHO cells are not only relevant for downstream quality assessment, but may also be required much earlier in the drug discovery pipeline. In this context, detailed structural studies rely on high-resolution, site-specific information that can only be obtained through resonance assignment. Taken together, these considerations highlight the need for selective isotopic labeling ^13^C of methyl groups, which provides the means to achieve reliable signal assignment and thus extends the impact of NMR from early-stage drug discovery to routine quality control.

For approximately two decades, selective methyl labeling has indeed become an essential strategy for studying high molecular weight proteins by NMR, offering enhanced sensitivity and resolution through targeted incorporation of ^13^CH₃-amino acids. In bacterial expression systems, these labeling schemes, combined with perdeuteration, are well-established and highly efficient (Kay and Gardner 1997; Goto et al. 1999; Tugarinov et al. 2006; Gans et al. 2010; Mas et al. 2013; Ayala et al. 2012; Kerfah et al. 2015), enabling detailed studies of large proteins and protein complexes. More recently, such approaches have been adapted to yeast with a moderate success (Clark et al. 2018; Miyazawa-Onami et al. 2013; Zhang et al. 2017; Suzuki et al. 2018), allowing improved structural investigations in systems closer to native folding and post-translational modifications. However, in more complex eukaryotic expression systems (namely insect and to a greater extent mammalian cells, which are the gold standard for producing therapeutic glycoproteins like mAbs), methyl labeling and perdeuteration remain technically challenging and underdeveloped. Although a few studies have reported successful methyl labeling in insect (Kofuku et al. 2012; Nygaard et al. 2013; Kleist et al. 2019; Korczynska et al. 2018; Solt et al. 2017) and HEK293 expression systems (Barbieri et al. 2016), most of these have focused on methionine residues. This is largely due to the commercial availability and affordability of ^13^CH₃-methionine, the use of methionine-depleted media in structural biology and the flexibility of its side chain, which helps mitigate relaxation effects in large proteins. Extending methyl labeling to branched-chain amino acids (BCAAs) such as isoleucine, leucine and valine remains challenging in eukaryotic systems. While Rosati et al. (2024) Röβler et al. (2024) showed that α-ketoisovalerate (KIV) can be efficiently used to label valine methyl groups in mammalian cells, the use of non-stereospecific KIV leads to incorporation of ^13^CH₃ probes into both valine methyl groups, resulting in two signals per valine. For applications requiring stereospecific valine labeling, particularly in large systems such as antibodies, the labeled amino acid remains necessary, as the stereospecific *pro-S* KIV precursor is unstable and partially epimerizes to the pro-R form, causing residual *pro-R* valine labeling. Similarly, Rosati et al. (2024) showed that α-ketoisocaproate can be used to label both methyl groups of leucines, whereas Dubey et al. (2021) reported a synthesis route for stereospecifically labeled leucine compatible with commonly used eukaryotic expression systems.

Achieving sufficient spectral quality also requires selective deuteration to minimize dipolar ¹H–¹H relaxation, particularly in large proteins. This can be done either by supplementing media with perdeuterated amino acids (Kofuku et al. 2014, 2018) although their use often leads to reduced expression yields, or by engineering partial deuteration of the methyl bearing amino acid side chains. Such strategies have been shown to markedly improve both signal resolution and intensity. For instance, Yanaka et al. (2018) demonstrated that stereospecific labeling and side-chain deuteration of Leu-δ_2_ groups in a full-length IgG2b antibody yielded well-resolved spectra, enabling the detection of all 42 expected methyl signals from the Fab fragment. Similarly, Dubey et al. (2021) showed that selective deuteration of leucine side chains improved spectral resolution by 30 to 50%.

Together, these studies underscore the importance of integrating selective methyl labeling with tailored deuteration schemes to extend the applicability of NMR spectroscopy to complex therapeutic proteins expressed in mammalian systems, while also highlighting the need for further methodological development to overcome current limitations in amino acid availability, especially for large protein systems.

In this work, we developed a ^13^C-labeling strategy selectively targeting the methyl groups of BCAAs (regioselective for isoleucine-δ_1_ and stereospecific for valine-γ-*pro-R* and leucine-δ-*pro-S*) as well as those of alanine, methionine, and threonine. This labeling scheme was implemented in CHO cells, the standard platform for clinical-grade antibody production. Using this strategy, we successfully expressed, at yields compatible with NMR requirements, a therapeutic antibody directed against lysosome-associated membrane protein 1 (LAMP1), a glycoprotein implicated in lysosomal function and frequently overexpressed in various cancer types (Agarwal et al. 2014; Alessandrini et al. 2017; Cameron et al. 2020). Anti-LAMP1 antibodies have shown promise as oncology drug candidates, making them a relevant and structurally challenging test case. High-resolution SOFAST-methyl-TROSY spectra revealed a dense array of well-dispersed signals, demonstrating the capacity of our labeling approach to probe mAb conformational landscapes in a biophysically and biologically relevant context. Using assignment transfer from the corresponding Fab and Fc fragments enabled assignment of 84% of the methyl signals in the full antibody, improving spectral interpretability and helping bridge structural biology and biopharmaceutical quality control.

## Materials and methods

### Enzymatic synthesis of L-isoleucine and L-valine

U-(^2^H,^15^N), (^13^CH_3_)^δ1^-isoleucine and U-(^2^H,^15^N), (^13^CH_3_)-*pro-R*-valine were enzymatically synthesized *via* two parallel reaction cascades starting from (S)-2-(D_3_) aceto-2-hydroxy-(4-^13^C; 3,3-D_2_) butanoate and (S)-2-hydroxy-2-(D_3_)methyl-3-oxo-(2,4-^13^C_2_) butanoate (NMR-Bio), respectively. Reactions were carried out in 50 mM Hepes buffer (pH 7.3) prepared in D₂O, supplemented with 10 mM MgCl₂.

For U-(^2^H,^15^N), (^13^CH_3_)^δ1^-isoleucine synthesis, 60 mM (final concentration) of (S)-2-(D_3_) aceto-2-hydroxy-(4-^13^C; 3,3-D_2_) butanoate was incubated with recombinant ketol-acid reductoisomerase (KARI, 20 µM) and dihydroxyacid dehydratase (DHAD, 10 µM) in a single reaction tube, allowing sequential conversion to labeled (R)-2,3-dihydroxy-3-methylpentanoate ((2R,3R)-2,3-dihydroxy-3-(D_3_)methyl-(5-^13^C ; 2,4,4-D_3_) pentanoate) and subsequent dehydration to labeled (S)-3-methyl-2-ketopentanoate ((S)-3-(D_3_)methyl-2-oxo-(5-^13^C ; 3,4,4-D_3_) pentanoate). The reaction mixture also contained 0.5 mM NAD⁺ as KARI is a NADPH-dependent enzyme. To support NAD⁺/NADH cofactor regeneration and enhance reaction efficiency, formate dehydrogenase (FDH, 0.5–1.5 U/mL; Merck, catalog number 10837016001) was added along with 100 mM sodium formate. The reaction was monitored by NMR and typically completed after 4–5 h incubation at 37 °C.

Reductive amination was then initiated by adjusting the pH of the sample to 9.6 and adding 100 mM ammonium chloride and leucine dehydrogenase (LeuDH, 2 U/mL; Sorachim, catalog number LED-201). ^15^N ammonium chloride was used during the reductive amination step, although the resulting ^15^N labeling was not exploited in this study. Since LeuDH is a NAD⁺-dependent enzyme, the cofactor regeneration system (formate dehydrogenase with sodium formate and NAD⁺) was reintroduced as described above. L-isoleucine formation was completed after 5 h incubation at 37 °C.

In a similar way, U-(^2^H,^15^N), (^13^CH_3_) )-*pro-R*-valine was synthesized from 140 mM (final concentration) of (S)-2-hydroxy-2-(D_3_)methyl-3-oxo-(2,4-^13^C_2_) butanoate using the same enzymatic steps, reaction conditions and cofactor recycling system.

The sequential steps of both syntheses are illustrated in Figure S1 and detailed production and purification protocols for KARI and DHAD are provided in the Supplementary Material.

All reactions were monitored by NMR and stopped by heat inactivation (20 min at 80 °C), followed by centrifugation (20 min at 4000 rpm). The resulting amino acids were purified on a 10 mL ProPack^™^ Rxn CX cation exchange resin (Waters- catalog number: 186004569) by gravity flow. The resin was equilibrated with 5 column volumes (CV) of methanol. Samples were diluted in 40 mL H₂O, acidified to pH 1.1 and loaded onto the column. After washing with 4 CV of methanol, amino acids were eluted with 4 CV of methanol containing 5% NH₄OH. Eluted fractions were pooled, dried by rotary evaporation and then resuspended in either H₂O or D₂O as required, prior to quantification by ¹H NMR.

### Expression of unlabeled anti-LAMP1 antibody in CHO cells

Stably anti-LAMP1-transfected CHO cells were grown in CDM4PERMAb medium (HyClone), supplemented with Cell Boost 7a and 7b (HyClone), 4 mM L-glutamine (Merck), 400 µg/mL hygromycin B Gold, and 400 µg/mL G418 (Invitrogen). For amplification purposes, cell cultures were maintained at 37 °C and 5% CO₂ in shake-flasks placed in orbital shaking incubators (200 rpm, 25 mm orbital diameter) and subcultured every 3–4 days by diluting back to 0.5 × 10⁶ cells/mL and to a viability superior to 95%. Antibody production was performed in CDM4PERMAb-based cultures in batch mode, also using shake-flasks, with an initial cell density of 1 × 10⁶ cells/mL for 4 days (or 1 × 10^7^ cells/mL for 2 days) under the same incubation conditions. Cell viability and density were monitored using trypan blue exclusion and manual counting with a Neubauer hemocytometer.

### Antibody purification and quantification

Supernatants were harvested by centrifugation (200 × g, 15 min), diluted 3-fold in 20 mM Tris, 20 mM NaCl, pH 7.4 (Buffer A) and purified by affinity chromatography on a 5 mL Hiscreen MabSelect Sure column (Cytiva) using a chromatography system. After column equilibration in Buffer A, the sample was loaded at 1 mL/min, followed by sequential washes with 5 column volumes (CV) of Buffer A, 2 CV of Buffer B (20 mM Tris, 1 M NaCl, pH 7.4) and another 5 CV of Buffer A. The antibody was eluted with 6 CV of Buffer C (100 mM glycine, 10 mM NaCl, pH 3.0) and fractions (1 mL) were neutralized by pre-adding 30 µL of 1 M Tris base pH 8.0 in collection wells.

Eluted fractions were pooled and concentrated using a 50 kDa MWCO Amicon ultrafiltration device (Merck) down to 0.5 mL, then subjected (when needed) to size-exclusion chromatography on a Superose 6 10/300 GL column (Cytiva) at 0.5 mL/min in 50 mM Tris, 150 mM NaCl, pH 7.5. Collected fractions were analyzed by SDS-PAGE and concentrated. Antibody concentration was determined by UV absorbance at 280 nm using a Nanodrop 2000 (Thermo Fisher) with ε = 223,400 M⁻¹·cm⁻¹ and a molecular weight of 144,724 Da.

### Isotopic labeling for NMR

For amplification purposes, anti-LAMP1-transfected CHO cells were grown in CDM4PERMAb medium (HyClone), supplemented with Cell Boost 7a and 7b (HyClone), 4 mM L-glutamine (Merck), 400 µg/mL hygromycin B Gold, and 400 µg/mL G418 (Invitrogen). Cell cultures were maintained at 37 °C and 5% CO₂ in shake-flasks placed in orbital shaking incubators (200 rpm, 25 mm orbital diameter) and subcultured every 3–4 days by diluting back to 0.5 × 10⁶ cells/mL. Once the required cell count was reached, cells were harvested by centrifugation (200 g, 10 min), washed twice with CDM4PERMAb amino acid-depleted medium (Hyclone) and inoculated at 10 × 10⁶ cells/mL in depleted medium. The cultures were maintained for 16 h under the same incubation conditions (37 °C, 5% CO_2_, 200 rpm, 25 mm orbital diameter) for amino acid starvation.

A labeled medium was prepared by complementing CDM4PERMAb amino acid-depleted medium (Hyclone) with the 20 standard amino acids, according to the desired labeling scheme, at the concentrations defined in the last column of Table 1. The medium was buffered at pH 7.5 and filtered prior to use. The labeled valine and isoleucine were synthesized in-house as described above, whereas the other labeled amino acids were commercially available and can be purchased from various suppliers (Eurisotop, Maglab, Merck, NMR-Bio, etc.).

**Table 1:** Amino acid composition of the different supplementation mixes

	AAmix1 (concentration mg/L)	2* AAmix1 (concentration mg/L)	Non labeled AAmix1_OptimAb (concentration mg/L)	Labeled AAmix1_OptimAb (concentration mg/L)
Alanine	93,4	186,8	107,6	350 +L-cycloserine 10 mg/L
Arginine	183,4	366,8	95,7	95,7
Asparagine	260	520	117,9	117,9
Aspartic acid	300	600	142,4	142,4
Cysteine	46	92	66,1	66,1
Glutamic acid	356	712	152,6	152,6
Glutamine	400	800	156,7	156,7
Glycine	60	120	101,5	101,5
Histidine	50,4	100,8	72,0	72,0
Isoleucine	172	344	72,4	**250**
Leucine	92	184	223,9	**250**
Lysine	169	338	246,7	246,7
Methionine	208	416	27,3	**250**
Phenylalanine	222	444	112,9	112,9
Proline	122,4	244,8	181,3	181,3
Serine	118,4	236,8	297,2	297,2
Threonine	60	120	223,6	**250**
Tryptophan	24	48	86,4	86,4
Tyrosine	90	180	168,8	168,8
Valine	50,4	100,8	244,0	**250**
Total (g/L)	**3**,**1**	**6**,**2**	**2**,**9**	**3**,**6**

The first three columns correspond to the amino acid mixtures tested to optimize culture yields under unlabeled conditions, whereas the last column shows the mixture used to produce the methyl-labeled mAb. The concentrations of the six amino acids bearing methyl groups that were modified relative to the unlabeled AAmix1_optimAb are indicated in bold

Following the starvation step, cells were harvested and washed again with depleted medium. Antibody production was performed in shake-flaks in labeled medium, in batch mode, with an initial cell density of 1 × 10^7^ cells/mL for 48 h, under the same incubation conditions (37 °C, 5% CO_2_, 200 rpm, 25 mm orbital diameter).

The same procedure was applied for all labeling schemes, except for alanine, where L-cycloserine (10 mg/L) was added to control intracellular alanine synthesis throughout both the starvation and production phases.

For NMR experiments, antibody expression was performed in CDM4PERMAb medium devoid of amino acids and supplemented with a custom mixture of labeled amino acids and selective antibiotics. The labeling strategy involved selective methyl-group labeling using isotopically enriched amino acids: U-[¹H], [¹³CH₃]ε-Met (Sigma-Aldrich), 2-(D)-3-(¹³C)-L-Ala (NMR-Bio), 2,3-(D₂)-4-(¹³CH₃)-L-Thr (NMR-Bio), 2,3,3,4,5,5,5-(D₇)-*pro-S*-¹³CH₃-L-Leu (Sigma-Aldrich) as well as ¹⁵N-2,3,4,4,4-(D₅)-*pro-R*-¹³CH₃-L-Val and ¹⁵N-2,3,4,4-(D₄)-3-methyl-(D₃)-5-¹³CH₃-L-Ile-δ₁ (produced using the protocol described above).

### NMR spectroscopy

For NMR experiments, samples were buffer-exchanged into NMR buffer (50 mM MES, 100 mM NaCl, pH 6.5 10% D₂O). NMR experiments were conducted at 35 °C on 600, 850 or 950 MHz Bruker Avance spectrometers equipped with cryogenic probes. 2D [¹H,¹³C] SOFAST-methyl_TROSY spectra (Amero et al. 2009) were recorded focusing on the methyl region. All relevant SOFAST experiment parameters are provided in Supplementary Table S1. Data processing was performed using TopSpin version 4.1 (Bruker). All NMR measurements were conducted using 3 mm Shigemi tubes in the same buffer conditions.

### Mass spectrometry analysis

For MS-based quantification of labeling efficiency, antibodies were expressed using the same protocol with selective isotopic labeling of a single methyl-containing amino acid per sample. Labeling was achieved using U-[¹H, ¹³C, ¹⁵N]-Val (Merck), -Thr (Merck), -Leu (Eurisotop), and -Ile (Martek Isotope) and U-[¹H], [¹³CH₃]^ε^-Met (Merck). Alanine-labeled samples were prepared with U-[¹H], [¹³CH₃]^β^-Ala (Eurisotop) and 10 mg/L L-cycloserine.

Prior to MS intact mass analysis, samples were deglycosylated and reduced using 12.5 µL of Rapid PNGase F buffer (Roche) at 75 °C for 10 min, followed by 1 µL of dithiotreitol (DTT) at 100 mM at 50 °C for 15 min in 50 µL antibody solutions (0.5 mg/mL). Mass spectra were acquired on an EXION LC / X500B QTOF-MS system (AB Sciex) (Sciex).

Incorporation efficiency for samples analyzed at the intact protein level was calculated from observed mass shifts of the heavy and light chains using the formula:

$$\begin{aligned}&\:Incorporation\:\left(\%\right)\\&=1-\frac{Observed\:mass-theoretical\:mass}{Mass\:increment\:per\:labeled\:residue\:\times\:\:Number\:of\:residue}\\&\times\:100 \end{aligned}$$

For samples treated in a peptide mapping workflow, 100 µg of antibody were buffer-exchanged, digested with immobilized trypsin beads, reduced, alkylated, and quenched prior to LC-MS analysis. Incorporation levels were calculated from peptide MS intensities, and averages and standard deviations were determined across the analyzed peptides or chains.

## Results

### Tailored enzymatic synthesis of labeled Val and Ile for eukaryotic expression systems

**Fig. 1:**
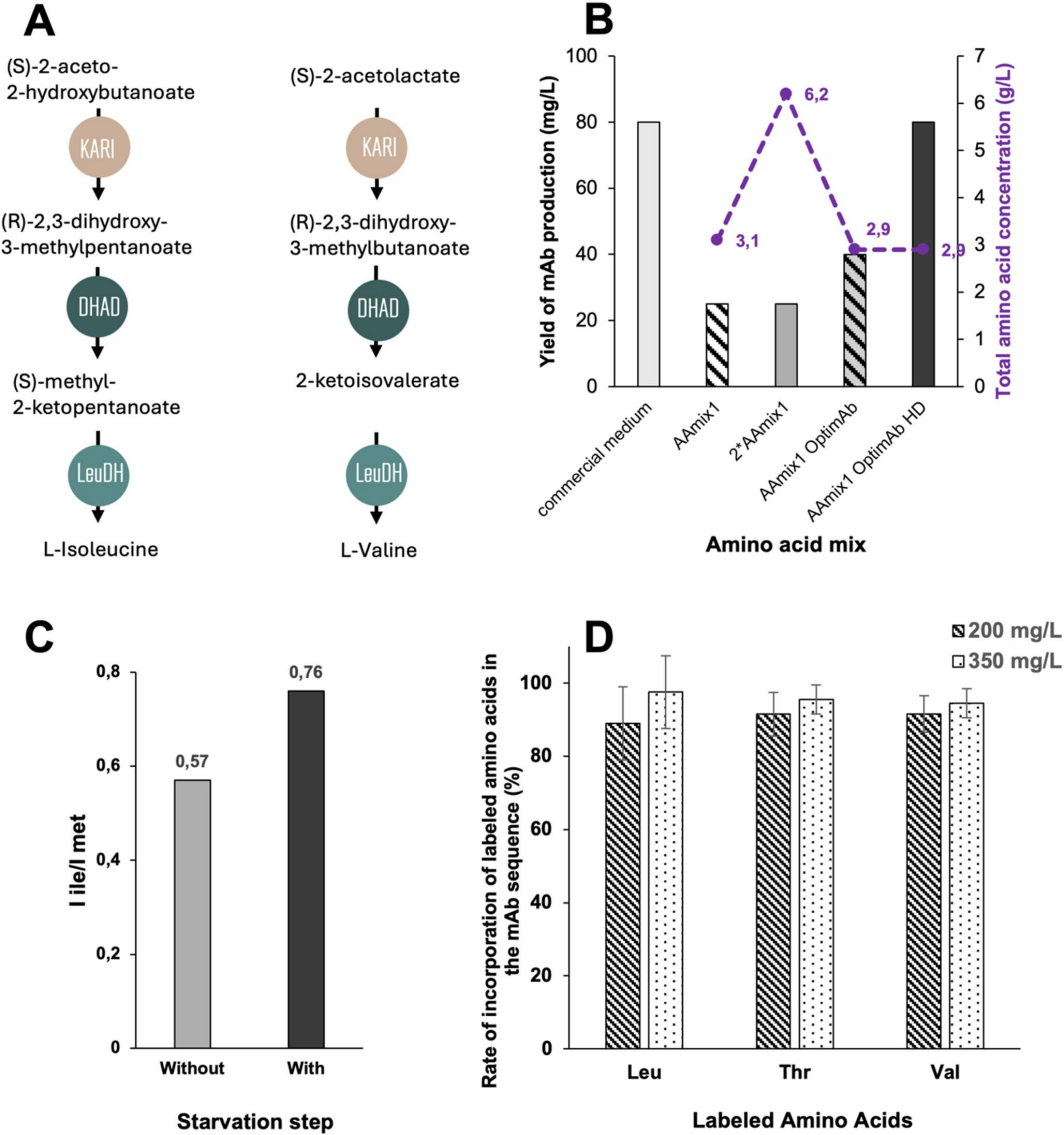
Optimization of amino acid supplementation strategies to improve selective labeling and expression yield of the anti-LAMP1 antibody in CHO cells. **A**) Schematic overview of the synthetic pathways of isoleucine and valine. The key enzymes involved in each step are indicated in colored bubbles. **B**) Combined representation of anti-LAMP1 antibody production yield (left Y-axis, bars) and total amino acid concentration in the culture medium (right Y-axis, line) under different supplementation conditions (HD: High cell Density). Each mAb sample was produced only once; therefore, no error bars are shown. **C**) Ratio of the mean peak intensities of isoleucine to methionine residues in 2D NMR spectra, comparing cultures with or without a 16-hour growth starvation step. Ile was supplemented at 100 mg/L and Met at 1 g/L. Labeled mAb samples were generated once; therefore, error bars are not shown. **D**) Amino acid incorporation rates (%) of Leu, Thr and Val supplemented at 200 mg/L or 350 mg/L into the anti-LAMP1 antibody sequence, as determined by mass spectrometry

In bacterial systems, selective methyl labeling of branched-chain amino acids is typically achieved by supplementing isotopically labeled α-ketoacid precursors which are efficiently converted intracellularly into isoleucine, valine and leucine. This approach allows straightforward incorporation of ^13^C-methyl groups into the desired residues (Fig. 1A). In eukaryotic expression systems, only the use of α-ketoisovalerate/isocaproate precursors is feasible to label methyl groups of valine/leucine residues; however, high-molecular-weight proteins require stereospecific methyl labeling to reduce spectral overlap by restricting labeling to a single methyl group of valine and leucine. While stereospecific labeling using α-ketoacid precursor is possible, it may be affected by epimerization of stereospecifically labeled ketoisovalerate under certain conditions, resulting in residual *pro-R* (or *pro-S*) labeling. Our data indicate that epimerization of the stereospecific pro-S ketoisovalerate precursor is relatively slow in vitro, occurring on a timescale of several days under standard conditions, but still sufficient to compromise stereochemical purity upon storage or prolonged handling. To preserve stereochemical integrity, the ketoacid intermediate can either be stored at − 80 °C to significantly slow epimerization or directly converted into the corresponding L-valine, the latter strategy being adopted in this work.

To address this, and to enable the methyl-specific labeling of isoleucine residues, we developed an efficient in vitro enzymatic synthesis protocol yielding regio- and stereoselectively ^13^CH₃-labeled amino acids suitable for eukaryotic expression and NMR studies of large proteins such as antibodies.

First, to produce the enzymes required for the enzymatic synthesis, recombinant ketol-acid reductoisomerase (KARI) and dihydroxyacid dehydratase (DHAD) were expressed in *E. coli* and purified, yielding 300 and 600 mg/L of enzyme, respectively. DHAD, a Fe-S cluster-containing enzyme, was tested under both aerobic and anaerobic (glovebox) conditions, however no significant improvement in catalytic performance was observed under anaerobic conditions during short-term assays. All subsequent reactions were therefore carried out in air with minimal handling to preserve enzyme integrity.

Using these purified enzymes, U-(^2^H,^15^N), (^13^CH_3_)^δ1^-Isoleucine and U-(^2^H,^15^N), (^13^CH_3_) *pro-R* valine were synthesized via a three-step enzymatic cascade. The first two steps, catalyzed by KARI and DHAD, were performed sequentially in the same buffer at pH 7.3. For isoleucine or valine, KARI catalyzes the rearrangement and reduction of the 2-aceto-2-hydroybutanoate precursor to its corresponding dihydroxy acid in an Mg²⁺- and NAD(P)H-dependent reaction (Dumas et al. 2001), while DHAD dehydrates the resulting dihydroxy acid to the corresponding α-keto acid (Carsten et al. 2015). The final reductive amination step, catalyzed by leucine dehydrogenase (LeuDH) at pH 9.6, yielded the amino acid with stereospecific ¹³C-methyl incorporation. Cofactor regeneration was implemented using formate dehydrogenase (FDH) to maintain NADH levels throughout the cascade.

Following enzymatic synthesis, the labeled amino acids were purified by cation exchange chromatography to remove residual precursors and by-products, preventing cytotoxicity during CHO cell culture. Overall molar yields were approximately 70–75% for δ₁-isoleucine and *pro-R*-valine. ¹H 1D NMR spectra confirmed the formation of Ile-δ₁-Ile and Val-*pro-R* with expected chemical shifts and signal intensities (Figs. S2-S3, Supplementary Data). These amino acids were then supplemented into a reconstituted chemically defined medium to produce a methyl-labeled anti-LAMP1 antibody in CHO cells.

### Improved antibody expression in CHO cells through amino acid mix optimization and high-density culture for NMR applications

The first critical step in enabling isotopic labeling of therapeutic antibodies in mammalian cells was to establish a reliable and efficient CHO cell culture protocol for the production and purification of the anti-LAMP1 monoclonal antibody. Rich commercial media used for therapeutic production support high levels of expression but contain large amounts of unlabeled amino acids. For isotopic labeling, media containing unlabeled amino acids or supplements are unsuitable, as they dilute labeled amino acids leading to lower isotope incorporation rates. We therefore used amino acid–free media supplemented with optimized amino acid mixtures, balancing yield and labeling efficiency.

To design the amino acid mixture for isotope labeling in CHO cells, we adapted the formulation of Gossert et al. (2011), reducing it fivefold to account for the markedly lower amino acid requirements of mammalian cultures compared with insect cell systems. We tested this formulation (AAmix1 - see recipe in Table 1) in CHO cells by supplementing it into a chemically defined, amino acid–depleted medium (Fig. 1B). Under these conditions, the yield reached 25 mg/L. Doubling the concentration of the mix (2×AAmix1) did not lead to any significant improvement in yield. This suggests that, although the overall amino acid concentration was increased from approximately 3 to 6 g/L, certain amino acids may still remain limiting for optimal antibody expression in CHO cells.

These observations led us to consider whether the amino acid composition itself, rather than the total amount, might be suboptimal for CHO expression of our specific antibody. Analysis of the anti-LAMP1 sequence guided the design of a tailored amino acid mixture to better reflect the composition of the recombinant mAb. Based on this, we developed a new formulation (AAmix1_OptimAb, Table 1), in which the relative molar concentrations of individual amino acids were adjusted according to the composition of the anti-LAMP1 antibody, reflecting the relative molar fraction of each residue in its sequence (Table S2). The total amino acid content was kept to approximately 3 g/L, similar to that of AAmix1. This approach was inspired by the work of Carrillo-Cocom et al. (2015), who demonstrated that amino acid consumption in CHO cells varies according to the amino acid composition of the overexpressed protein.

Supplementing the culture medium with this customized mixture improved antibody yields to 40 mg/L, approximately a twofold increase compared to the initial AAmix1 formulation, although still lower than the yields obtained with full commercial media (80 mg/L). This suggests that the amino acids present at higher concentrations in AAmix1_OptimAb compared to AAmix1 play a particularly important role in supporting anti-LAMP1 antibody production.

Further optimization of isotope labeling in CHO cells involved timing the addition of labeled amino acids to align with the phase of active protein synthesis. During the lag and exponential growth phases, nutrients are primarily used for cell proliferation rather than protein synthesis. To avoid unnecessary isotope consumption during cell growth, we implemented a high-cell-density culture strategy, initiating cultures at 1 × 10⁷ cells/mL (the maximum viable density achievable in flasks during cell passage) instead of 1 × 10^6^ cells/mL. Under these conditions, antibody production proceeded for 48 h before cells entered apoptosis, yielding levels (80 mg/L) comparable to those obtained with commercial rich media (Fig. 1B). This allowed recovery of 4 mg of purified anti-LAMP1 antibody from 50 mL of culture, sufficient for NMR analysis.

### Enhanced methyl labeling efficiency in CHO cells through cell starvation

In our standard protocol for isotope labeling, cells were initially cultured in commercially available, unlabeled amino acid–rich medium to allow sufficient biomass accumulation. Before labeling, they were harvested by centrifugation and washed twice with amino acid-depleted medium to eliminate residual unlabeled amino acids from the extracellular environment. The cells were then resuspended in a defined labeling medium containing the desired labeled amino acids. However, despite thorough washing, intracellular pools of unlabeled amino acids, accumulated during the growth phase, can persist and be used during protein synthesis, leading to isotopic dilution (Sitarska et al. 2015). To address this, a starvation step was introduced in which cells were incubated at high density (1 × 10⁷ cells/mL) in amino acid-free medium prior to labeling. In our system, starvation durations of 4, 8, and 16 h were evaluated, and 16 h was selected based on its ability to maintain > 90% cell viability.

For labeling, U-(²H,¹⁵N), (¹³CH₃) δ¹-Isoleucine was used at 100 mg/L to remain below excess levels and enable detection of differences between the two conditions, while ¹³CH₃-methionine was added in excess (1 g/L) as an internal reference. NMR analysis of anti-LAMP1 antibody produced with and without starvation revealed a significant improvement in labeling efficiency, with the Ile/Met peak intensity ratio increasing from 0.57 to 0.76, corresponding to a 1.3-fold labeling efficiency enhancement (Fig. 1C). Importantly, antibody yield remained high (79 mg/L), confirming that this starvation step enhances isotope incorporation without compromising protein production.

To quantify the incorporation of labeled amino acids in CHO-expressed antibodies, mass spectrometry (either at the intact protein level or using peptide mapping) was performed on samples in which only one methyl-containing amino acid type was labeled at a time. This strategy allowed quantitative measurement of incorporation yield for each residue type without overlapping mass contributions. In contrast to NMR samples, non-deuterated [U-¹³C, ¹⁵N]-labeled amino acids were used to prevent mass heterogeneity arising from partial Cα-deuterium/hydrogen exchange catalyzed by cellular transaminases during protein synthesis (Sitarska et al. 2015) and to maximize the overall isotopic mass increment by uniformly labeling all carbon atoms of the considered amino acids. Incorporation rates were determined by measuring the mass shift of intact heavy and light antibody chains. For valine, threonine and leucine, supplementation with 200 mg/L of each labeled amino acids resulted in efficient incorporation with labeling levels approaching saturation around 90%. Increasing the concentration to 350 mg/L did not further improve incorporation, indicating that maximal uptake efficiency had been reached under these conditions (Fig. 1D). For methionine and isoleucine, incorporation was estimated to be greater than 84%, in agreement with the levels observed for the other residues shown in Fig. 1D. Based on these results, an intermediate concentration of 250 mg/L was selected for Methionine, Isoleucine, Valine, Leucine and Threonine, as reported in the last column of Table 1.

### Improved ¹³CH₃-alanine incorporation via transaminase inhibition

**Fig. 2:**
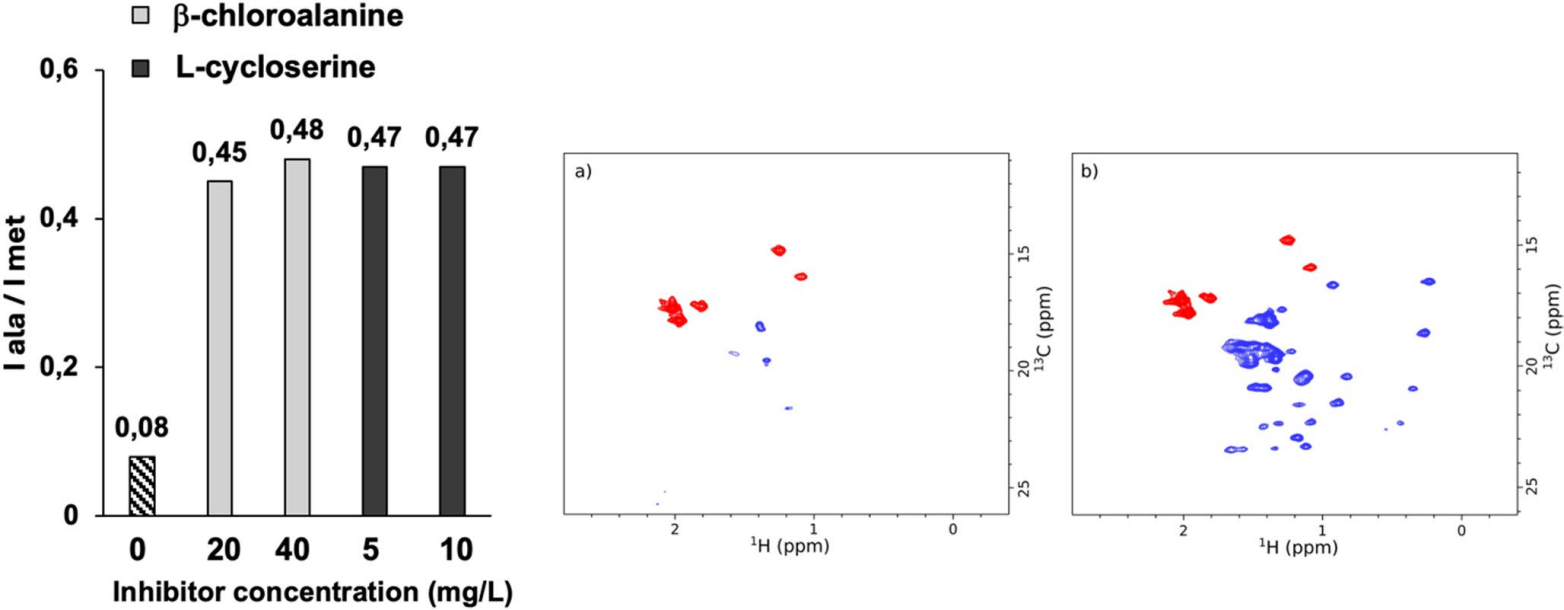
Alanine Transaminase Inhibitors Increase ¹H-¹³C NMR Signal Intensity of Alanine Residues **A**) Impact of alanine transaminase inhibitor type and concentration on the ¹H-¹³C NMR signal intensity of labeled alanine residues. CHO cells were cultured with U-[²H], [¹³C¹H₃]β-alanine and 1 g/L [¹³C¹H₃]ε-methionine, in the absence or presence of either β-chloroalanine (20 or 40 mg/L) or L-cycloserine (5 or 10 mg/L). Peak intensities of alanine signals were normalized to methionine signals. **B**) ¹H-¹³C SOFAST-methyl-TROSY spectra of U-[¹H], [¹³C¹H₃]ε-Met and U-[²H], [¹³C¹H₃]β-Ala labeled anti-LAMP1 IgG1 produced in CHO cells without (a) or with (b) 5 mg/L L-cycloserine. Methionine resonances are shown in red and alanine resonances in blue. Antibody concentrations were 40 µM (a) and 55 µM (b). Spectra were acquired at 35 °C on a 600 MHz NMR spectrometer equipped with a cryogenic probe. Each labeled mAb sample was produced only once; therefore, no error bars are shown

Among methyl-containing amino acids, alanine presents a specific challenge for isotopic labeling in CHO cells due to its efficient *de novo* synthesis from glucose *via* transamination, leading to significant isotopic dilution when supplemented in labeled form. For this reason, alanine was treated separately from other amino acids in our incorporation analysis. To suppress endogenous alanine biosynthesis and improve labeling efficiency, two alanine transaminase inhibitors (L-cycloserine and β-chloroalanine) were tested at various concentrations, as previously described for eukaryotic cell systems (Sitarska et al. 2015; Kofuku et al. 2018; Röβler et al. 2024) (Fig. 2A). NMR analysis of samples labeled with [¹³CH₃]-alanine revealed a 5.8-fold increase in alanine methyl peak intensity (with respect to a reference methionine signal) upon treatment with either inhibitor, regardless of compound identity or concentration, indicating a substantial reduction in unlabeled alanine incorporation (Fig. 2B). Based on these results, L-cycloserine at 10 mg/L was selected for further experiments. Incorporation efficiency was then quantified by mass spectrometry and reached 85% when 300 mg/L of labeled alanine was added to the culture medium.

### Selective methyl labeling enables residue-type discrimination in anti-LAMP1 antibody

Using the established labeling protocol, five distinct anti-LAMP1 mAb samples were produced in CHO cells, each selectively labeled on a single methyl-containing amino acid: alanine, threonine, isoleucine, leucine or valine. Methionine was included in all samples as a common reference label. For isoleucine, leucine and valine, labeling was regio- or stereo-specific (Ile-δ_1_, Leu-*pro-S*, Val-*pro-R*).

**Fig. 3:**
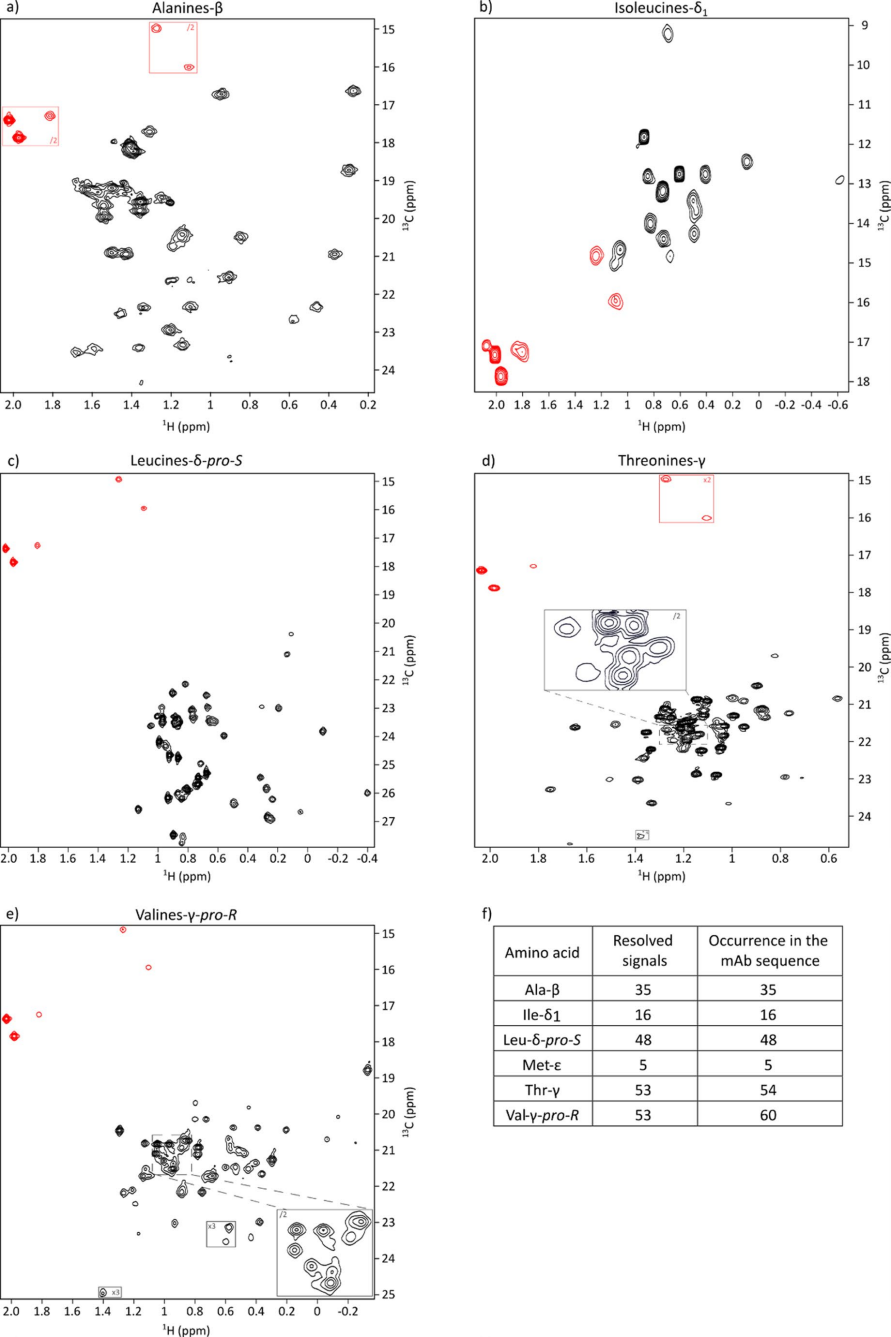
2D ¹H-¹³C SOFAST NMR spectra in the methyl region of the anti-LAMP1 antibody produced in CHO cells and labeled with [¹³CH₃]-methionine and: **a**) [U-²H, ¹³CH₃]-alanine, **b**) [U-²H, ¹³CH₃-δ1]-isoleucine, **c**) [U-²H, ¹³CH₃-proS]-leucine, **d**) [U-²H, ¹³CH₃]-threonine, **e**) [U-²H, ¹³CH₃-proR]-valine. Experiments were acquired at 35 °C on spectrometers equipped with cryogenic probes operating at ¹H frequencies of 850 MHz (**a**, **d**), 950 MHz (**c**, **e**), and 600 MHz (**b**). To better visualize peaks, multiple zoom levels were applied for spectra (**a**), (**d**) and (**e**) (red and black rectangles). Methyl peaks corresponding to methionine residues are shown in red on each spectrum. (**f**) Number of peaks counted on the spectra compared to number of peaks expected from the Anti-Lamp1 mAb sequence for each methyl bearing amino acids

¹H-¹³C SOFAST-methyl-TROSY spectra were acquired for each sample, revealing distinct and well-resolved cross-peaks for alanine (Fig. 3a), isoleucine (Fig. 3b), leucine (Fig. 3c) and methionine residues (Fig. 3 red peaks on the spectra). While some spectral overlap was observed for threonine (Fig. 3d) and valine (Fig. 3e) methyl groups, the overall resolution and signal-to-noise ratios were sufficient to support unambiguous residue-type classification as approximately 210 resolved peaks of the 218 expected signals (over 96%) can be seen on the spectra (Fig. 3f). These results confirm that the method enables detailed NMR analysis of CHO-expressed antibodies and provides a strong foundation for subsequent methyl resonance assignments.

### Structural integrity of methyl-labeled antibodies confirmed by NMR spectral comparison

Validation of the labeling strategy was achieved by overlaying 2D ^1^H-^13^C methyl SOFAST-methyl-TROSY NMR spectra of antibodies selectively labeled on each methyl-containing amino acid (Ile-δ^1^, Leu *pro-S*, Val *pro-R*, Met, Thr, and Ala) with the reference spectrum of the unlabeled antibody at natural isotopic abundance. The reference spectrum was recorded at 35 °C for 70 h on a Bruker 850 MHz spectrometer equipped with a cryogenically cooled probe, using a 0.25 mM sample. By comparison, the SOFAST-methyl-TROSY spectra of the selectively labeled antibodies were acquired with experimental times ranging from 1 to 4 h, depending on the sample, as detailed in Supplementary Table S1. As shown in Fig. S4, methyl resonances from labeled samples closely aligned with their natural abundance counterparts, indicating that isotope incorporation did not alter the mAb 3D structure. This spectral overlap demonstrates the structural integrity of the antibody under labeling conditions and confirms the reliability of the developed labeling protocol. Importantly, because valine, leucine and isoleucine residues were labeled on only one of their two methyl groups, the number of observable methyl signals in the labeled spectra was reduced compared to the natural abundance reference. This reduction led to decreased spectral crowding, thereby improving spectrum quality.

### Resonance matching between cell-free produced anti-LAMP1 Fab and Fc and CHO-derived mAb supports assignment transferability

While the results described above represent a major advance, demonstrating for the first time successful labeling of all six methyl-containing amino acids in a CHO-expressed antibody, they also highlight a key limitation. Without assignment of each NMR signal to specific atoms in the antibody’s 3D structure, it remains impossible to directly link spectral differences to functional or conformational changes. Such assignments would unlock the full structural potential of 2D NMR spectra, which remain underexploited in fingerprinting studies of mAbs. However, assigning methyl resonances in a 150 kDa mAb typically requires uniformly deuterated samples, which remains a major bottleneck for atomic-resolution NMR of full-length antibodies due to the toxicity of D₂O in eukaryotic cell cultures (Siegel et al. 1960).

To advance towards the assignment of methyl resonances in the full-length anti-LAMP1 monoclonal antibody expressed in CHO cells, a divide-and-conquer approach was adopted. As part of this strategy, the ^13^CH_3_ methyl labeled Fab fragment of the anti-LAMP1 antibody was previously produced in-house using a cell-free system, allowing the assignment of approximately 84% of its methyl resonances (Henot et al. 2026). A similar strategy was applied to the Fc fragment, leading to the assignment of 94% of its methyl groups (Vibert et al. 2026).

**Fig. 4:**
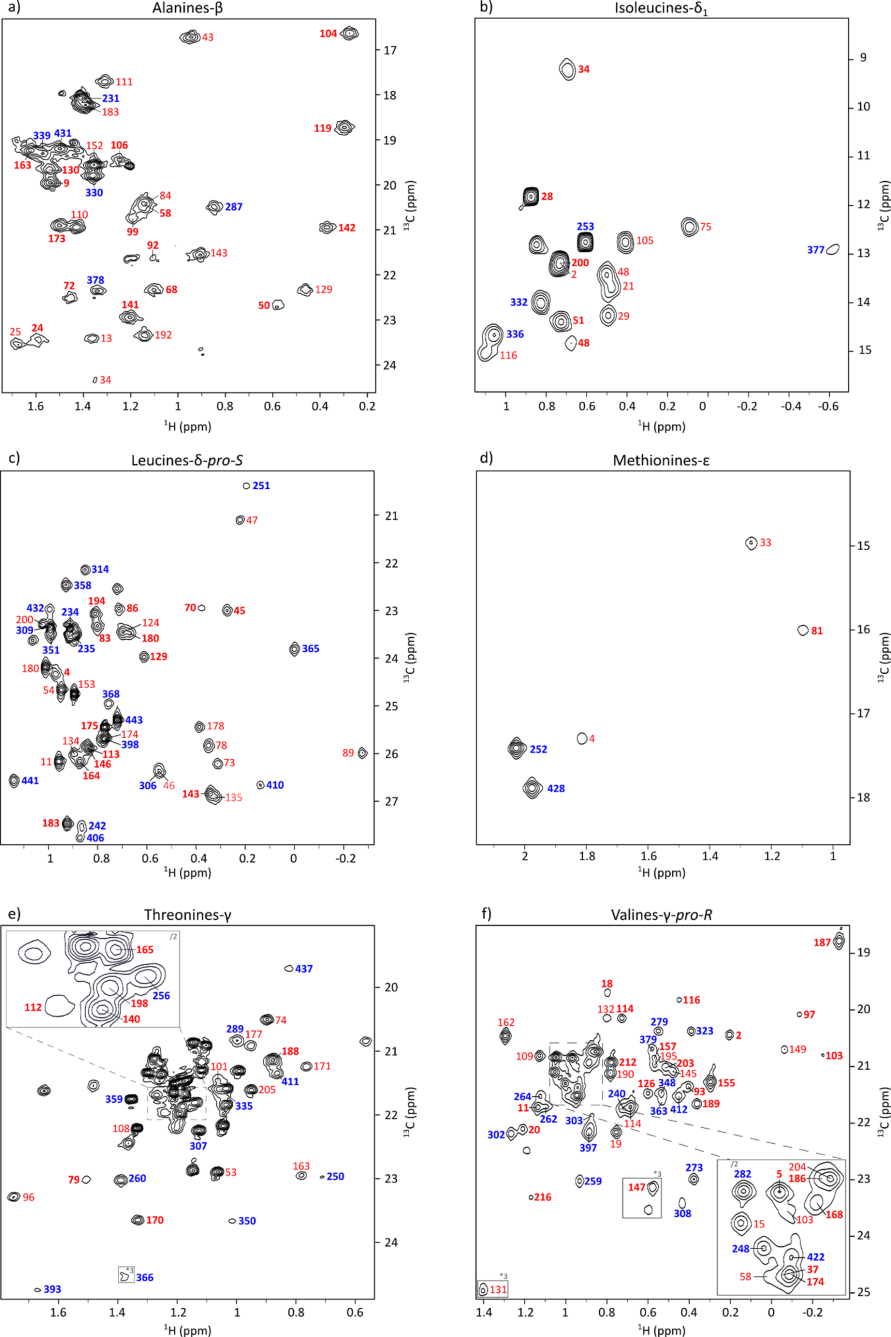
Assigned 2D ^1^H-^13^C SOFAST-methyl-TROSY spectra of the anti-LAMP1 mAb produced in CHO cells and specifically labeled on methyl groups. **(a)** mAb labeled on methionines-ε and alanines-β, zoom on alanines-β region. **(b)** mAb labeled on methionines-ε and isoleucines-δ_1_, zoom on isoleucines-δ_1_ region. **(c)** mAb labeled on methionines-ε and leucines-δ-*pro-S*, zoom on leucines-δ region. **(d)** mAb labeled on methionines-ε and valines-γ-*pro-R*, zoom on methionines-ε region. **(e)** mAb labeled on methionines-ε and threonines-γ, zoom on threonines-γ region. **(f)** mAb labeled on methionines-ε and valines-γ-*pro-R*, zoom on valines-γ region. Each assigned signal is annotated with the corresponding residue number: blue for the Fc fragment, red for the Fab fragment, bold for the heavy chain, and normal for the light chain. In panels **e** and **f**, contour levels were adjusted for some peaks, which are indicated with grey rectangles

The 2D ^1^H–^13^C SOFAST-methyl-TROSY spectra of this cell-free expressed Fab fragment were then overlaid with those of the full antibody produced in CHO cells to assess structural consistency and enable resonance transfer. As shown in Figure S5, the methyl signals from alanine, methionine, leucine-*pro-S*, isoleucine-δ_1_, threonine and valine-*pro-R* exhibited perfect spectral overlap between the Fab and the full-length mAb, confirming that the structure of the cell-free produced isolated fragment was preserved and allowing the transfer of assigned resonances (Fig. 4). For the Fc, the assignment transfer was less straightforward. Using the previously published results of Vibert et al. (2026), the methyl resonances of the non-glycosylated Fc fragment produced in the cell-free system were assigned, and this assignment was then transferred to the glycosylated Fc fragment obtained from the CHO-expressed mAb, before finally transferring the assignments to the full-length antibody.

This enabled successful assignment transfer for 97% of alanines, 100% of isoleucines-δ_1_, 96% of leucines-δ-*Pro-S*, 100% of methionines, 50% of threonines and 93% of valines-γ-*Pro-R*, resulting in an overall assignment of 84% of methyl containing residues in the mAb spectrum (table S3). The lower percentage of assigned threonines was due to their location in the Fab region, where most threonines were solvent-exposed and either exhibited mainly intermethyl NOE cross peaks with other unassigned threonines or showed no intermethyl NOE connectivity (Henot et al. 2026). Excluding threonines, the assignment transfer exceeds 95% of methyl-containing residues. This validates the use of cell-free expressed Fab and Fc as reliable surrogates for methyl resonance assignment in a full-length CHO-expressed antibodies.

## Discussion and conclusion

The development of therapeutic monoclonal antibodies as biopharmaceutical agents has highlighted the critical need for detailed structural and dynamic characterization techniques capable of assessing HOS at atomic resolution. NMR spectroscopy, especially methyl NMR based methods, has emerged as a powerful tool for probing large proteins and complexes in solution. However, the practical application of methyl labeling in full-length antibodies expressed in mammalian systems, such as CHO cells, has been significantly hampered by challenges inherent to the complexity of mammalian cell metabolism and the limited availability of isotopically labeled media.

In this study, we present an optimized approach for the selective isotopic labeling of the methyl groups in isoleucine, leucine, valine, alanine, methionine and threonine residues in an anti-LAMP1 antibody produced in CHO cells. Our strategy addresses several major obstacles previously limiting methyl labeling in mammalian systems. First, we developed an enzymatic synthesis route to obtain stereospecifically and regio-specifically ^13^C-labeled valine and isoleucine with high purity and isotopic enrichment. These stereospecifically labeled amino acids can also be obtained from suppliers of SAIL (Stereo-Array Isotope Labeling) amino acids (Kainosho et al. 2006; Kainosho and Güntert 2009) which are chemically synthesized. They were originally designed to minimize spectral overlap and to enhance NMR resolution and sensitivity by retaining, in a controlled manner, only a single proton at each atomic position. Since their introduction, SAIL chemists have broadened their protocols to achieve the synthesis of ^13^CH_3_- labeled amino acids with fully deuterated side-chains. Their application in mammalian expression systems is entirely feasible, however, it remains strongly dependent on market availability. We therefore developed this new protocol inspired by *E. coli* metabolic pathways to diversify the ^13^CH_3_-Val and Ile sources.

In our effort to synthesize BCAAs suitable for methyl labeling in mammalian systems, labeled leucine was not pursued, as its biosynthesis involves additional enzymatic steps, including oxygen-sensitive reactions, that are difficult to produce and handle in vitro, making an enzymatic route impractical. Nevertheless, alternative solutions for obtaining stereospecifically labeled leucine have been reported. Dubey et al. (2021) and Mallis et al. (2024) describe efficient synthetic strategies to produce ^13^C-labeled leucine with local deuteration scheme, offering an expanded repertoire of methyl-labeled BCAAs tailored for high-resolution NMR.

Our enzymatic protocol is modular and can be adapted to produce different labeling schemes of branched-chain amino acids (BCAAs) by varying the starting precursors, enabling the synthesis of δ_1_- or γ_2_-labeled isoleucine, *pro-R*- or *pro-S*-labeled valine, or other isotopomers (^13^CHD_2_…). Importantly, by using deuterated precursors and performing the reaction in D₂O-based buffer, the resulting amino acids carried stereospecific methyl labeling combined with side-chain deuteration, contributing to improved spectral resolution in NMR experiments. As previously demonstrated (Dubey et al. 2021; Yanaka et al. 2018), such tailored deuteration patterns are particularly advantageous for methyl-TROSY studies, reducing intra-residue ¹H–¹H dipolar relaxation and spectral overlap to enable high-resolution observation of large glycoproteins such as full-length antibodies.

In parallel, the cell culture medium was optimized to maintain high antibody titers while maximizing isotope incorporation, using tailored amino acid mixtures and controlled timing of labeled amino acid addition, minimizing dilution. A starvation step prior to labeling reduced intracellular pools of unlabeled amino acids, a major source of isotopic dilution. To overcome challenges in alanine methyl labeling (limited by its biosynthesis via transaminase activity) selective transaminase inhibitors were applied to block endogenous synthesis facilitating efficient incorporation of labeled alanine. These combined strategies resulted in a robust and reproducible platform achieving high isotopic incorporation rates (exceeding 85–90% for most methyl sites) without compromising antibody expression levels or quality.

Several studies have highlighted the importance of culture medium optimization to achieve high levels of isotope incorporation in mammalian cells for methyl-specific labeling. Mallis et al. (2024), for example, reported a tailored amino acid mixture based on Dulbecco’s modified Eagle’s medium (DMEM) composition that improved incorporation efficiency without compromising protein expression. Similarly, Röβler et al. (2024) demonstrated that careful design of the amino acid composition can significantly enhance methyl labeling in HEK293 cells. In their study, the amino acid content of the custom mix for 1 L of culture approximated the total amino acids present in 3 g of yeast extract, although residual amino acids in the base depleted medium and in added FBS make direct quantitative comparisons difficult. These studies, including our own, should be interpreted with caution, as differences in cell lines and culture densities can influence amino acid requirements and labeling efficiency. In the present work, we further show that antibody yield can be significantly improved when amino acid supplementation is tailored to the specific composition of the overexpressed protein. Collectively, these findings highlight that both the general metabolic demands of the host cells and the particular amino acid requirements of the target protein must be considered when optimizing media formulations. Overall, these studies underscore that systematic medium optimization is a key factor for successful isotope labeling of methyl groups in eukaryotic proteins, and that the accumulation of such knowledge is valuable for the community, as it enables high-resolution NMR studies of proteins expressed in eukaryotic systems.

Using our medium formulation, we obtained high-quality SOFAST-methyl-TROSY NMR spectra, exhibiting excellent resolution and sensitivity, with well-dispersed resonances clearly assignable to each methyl type. A key advantage of the high incorporation efficiency achieved is the dramatic reduction in acquisition times. Previous reports on unlabeled antibodies at natural isotopic abundance cite acquisition times around 12 h on a 900 MHz spectrometer at 50 °C for a mAb concentration of 250–300 µM (Arbogast et al. 2015). In stark contrast, selectively methyl-labeled antibodies produced in CHO cells yielded similar or better spectral information in less than 2 h at lower concentrations (50–100 µM), illustrating a remarkable gain in sensitivity and efficiency.

Overlaying spectra of the full-length labeled antibodies with those of cell-free expressed Fab and Fc fragments allowed unambiguous resonance assignment transfer and confirmed structural consistency between antibody fragments and intact IgG molecules. This is particularly valuable as it enables resonance assignment strategies developed for smaller, more tractable fragments to be extended to the full-length therapeutic molecule, facilitating comprehensive structural studies. This methodological advance overcomes a key limitation in the application of solution-state NMR to mammalian-expressed antibodies. It bridges the gap between structural studies conducted on bacterial or cell-free expressed antibody fragments and the need to analyze fully glycosylated, post-translationally modified and clinically relevant antibodies produced in industrially relevant mammalian cells. Consequently, it offers an unprecedented opportunity to study antibody conformational dynamics, domain interactions and subtle local structural perturbations that can arise from manufacturing variations, formulation stresses, or antigen binding.

Importantly, given that resonance assignment transfer between Fab fragments of IgG1 antibodies has been demonstrated (Henot et al. 2026), this strategy could be expanded to full-length mAbs sharing conserved domains. Building a comprehensive library of assigned antibody structures would ultimately allow the transfer of assignments to new antibodies without the need for isotope labeling, greatly streamlining and accelerating structural characterization workflows. This is particularly relevant since, although stable isotope labeling offers clear practical advantages for NMR, its application in large-scale pharmaceutical production remains limited by stringent regulatory constraints that currently preclude routine use of isotope-labeled amino acids.

Looking ahead, this platform could enable advanced antibody fragment screening, epitope mapping and the structural analysis of complex biopharmaceuticals such as antibody-drug conjugates and bispecific antibodies. Combined with complementary biophysical and computational techniques, these NMR methods promise comprehensive characterization of antibody therapeutics, ultimately guiding next-generation drug design and quality control to improve patient safety and clinical efficacy.

## Supplementary Information

Below is the link to the electronic supplementary material.

Supplementary Material 1

## Data Availability

The datasets and NMR spectra generated during the current study have been deposited in the Zenodo repository and are available at 10.5281/zenodo.19203174.
