# Supplementary material for: Methyl-specific NMR of therapeutic antibodies: cost-effective isotopic labeling strategies in CHO cells for high-resolution structural characterization
Source: J Biomol NMR. 2026 Apr 18;80(1):13. doi: 10.1007/s10858-026-00492-3 (PMC13090208; doi:10.1007/s10858-026-00492-3)
Supplement: Supplementary file 1 — Supplementary Material 1 [file 10858_2026_492_MOESM1_ESM.pdf]

**Methyl-Specific NMR of Therapeutic Antibodies: Cost-effective Isotopic Labeling Strategies in CHO Cells for High-Resolution Structural Characterization**

Rida Awad [a#], Arthur Giraud [a,b,c#], Béatrice Vibert [b,c], Séverine Clavier [b], Hélène Le Borgne [b], Maçon Laetitia [b], Muhr-Naninck Anaïs [b], Seguin-Huet Stéphanie [b], Mothes Benoit [b], Pierre Gans [c], Oriane Frances [b*], Jérôme Boisbouvier [c*] Elodie Crublet [a*]

[a] *NMR-Bio, 225 Route de Bivan 38470 L'Albenc, France.*
[b] *Sanofi Research & Development, 94403 Vitry-sur-Seine, France.*
[c] *Univ. Grenoble Alpes, CNRS, CEA, Institut de Biologie Structurale (IBS), 71 Avenue des Martyrs, F-38044 Grenoble, France.*

*# These authors contributed equally to this work*

** Correspondence to be addressed to crublet@nmr-bio.com, Oriane.Frances@sanofi.com or jerome.boisbouvier@ibs.fr*

# Supplementary information

**Supplementary methods** : Expression and purification of KARI and DHAD

**Figure S1.** Enzymatic pathways for the synthesis of U-($^2$H,$^{15}$N),($^{13}$CH$_3$)-$\delta_1$-isoleucine and U-($^2$H,$^{15}$N),($^{13}$CH$_3$)-*pro-R*-valine

**Figure S2** : Superposition of three 1D $^1$H NMR spectra recorded under identical conditions

**Figure S3** : Superposition of three 1D $^1$H NMR spectra recorded under identical conditions

**Figure S4** : Overlay of the 2D SOFAST-methyl-TROSY NMR spectra recorded on the methyl-labeled anti-LAMP1 antibody and the non-labeled anti-LAMP1 antibody expressed in CHO cells

**Figure S5** : Overlay of 2D $^1$H-$^{13}$C SOFAST-methyl-TROSY spectra of the anti-LAMP1 Fab fragment expressed in a cell-free system with those of the mAb expressed in CHO cells

**Table S1:** Experimental parameters of the 2D [$^1$H,$^{13}$C] SOFAST-methyl-TROSY experiments.

**Table S2** : Amino acid composition adjusted in AAmix1_OptimAb based on antibody sequence

**Table S3** : Number of assigned resonances on the spectra of the anti-LAMP1 mAb produced in CHO cells and specifically labeled on methyl groups

**Anti-LAMP1 sequence**

**Expression and purification of KARI and DHAD**

KARI was expressed from pET21 vector in *E. coli*. Cultures (2 L Terrific Broth (TB) + 5 mM $MgSO_4$) were induced at $OD_{600}$ = 2.5 with 1 mM IPTG for 3 h at 37 °C. Cells were lysed in 50 mM HEPES pH 8.0, 10% glycerol, 0.1% Tween 20, supplemented with DNase I, by sonication and clarified by centrifugation (30 min, 40,000 g). Purification was performed by IMAC (elution with 500 mM imidazole), followed by desalting on HiPrep 26/10 into perdeuterated buffer (50 mM HEPES pH 7.3, 10 mM $MgCl_2$, 10% glycerol, 0.1% Tween 20 in $D_2O$).

DHAD was expressed from pET28 vector in *E. coli.* Cultures (2 L TB + 5 mM $MgSO_4$, 1 mg/L $FeSO_4$) were induced at $OD_{600}$ 2.5 with 1 mM IPTG overnight at 20 °C. Cells were lysed in 50 mM Tris pH8 10 mM MgCl2 (TM8) buffer by sonication and clarified as above. Proteins were purified by IMAC (elution in TM8 buffer with 500 mM imidazole) and desalted on HiPrep 26/10 into TM8 prepared in $D_2O$.

Both enzymes were concentrated to 0.5 mM, aliquoted and stored at -70 °C.

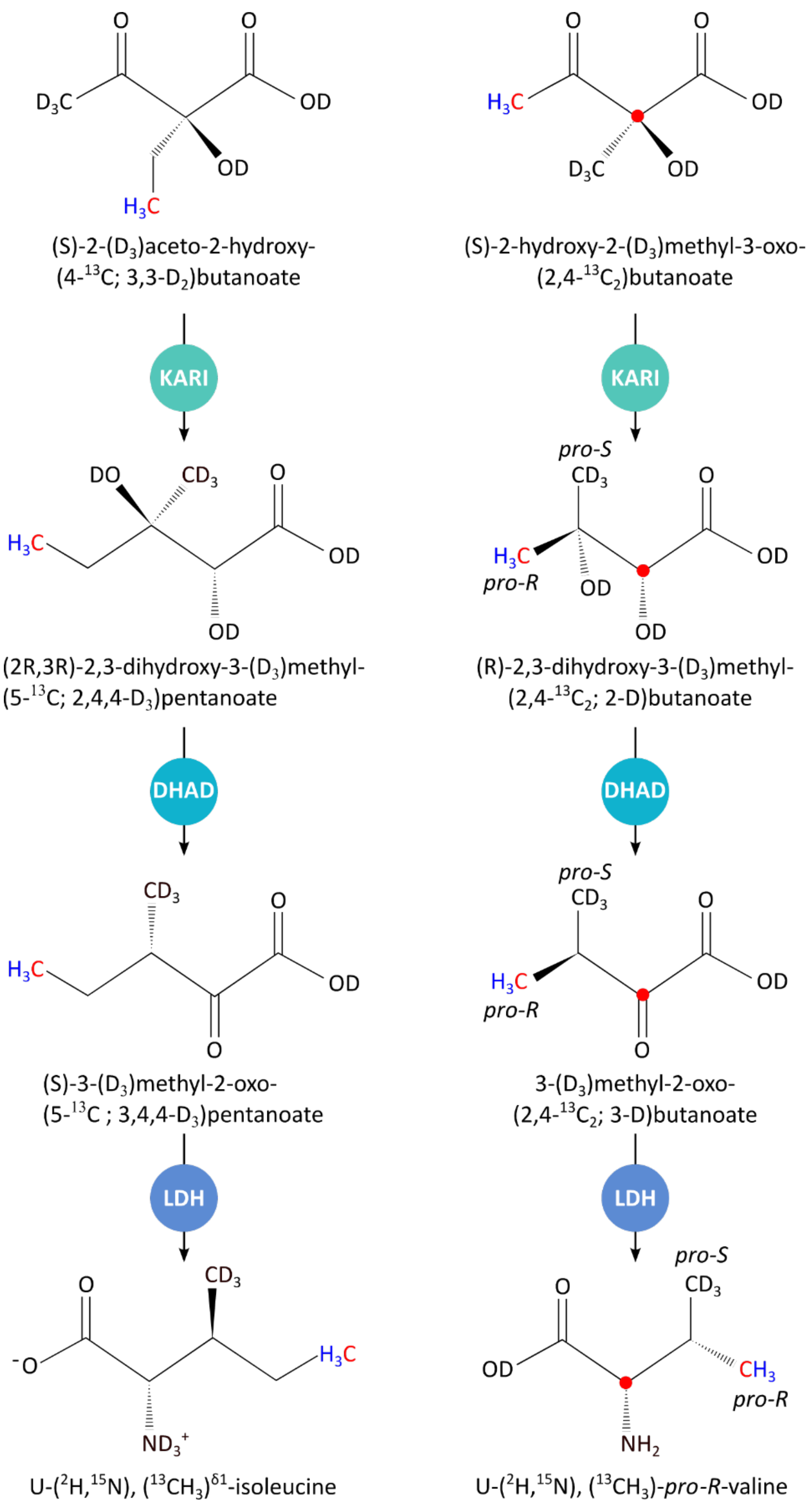


**Figure S1 : Enzymatic pathways for the synthesis of U-($^{2}$H,$^{15}$N),($^{13}$CH$_3$)-δ$_1$-isoleucine and U-($^{2}$H,$^{15}$N),($^{13}$CH$_3$)-*pro-R*-valine**, showing intermediates, labeling positions, and cofactor recycling steps. Labeled carbon atoms are indicated in red and the protons of the $^{13}$CH$_3$ methyl groups are shown in blue.

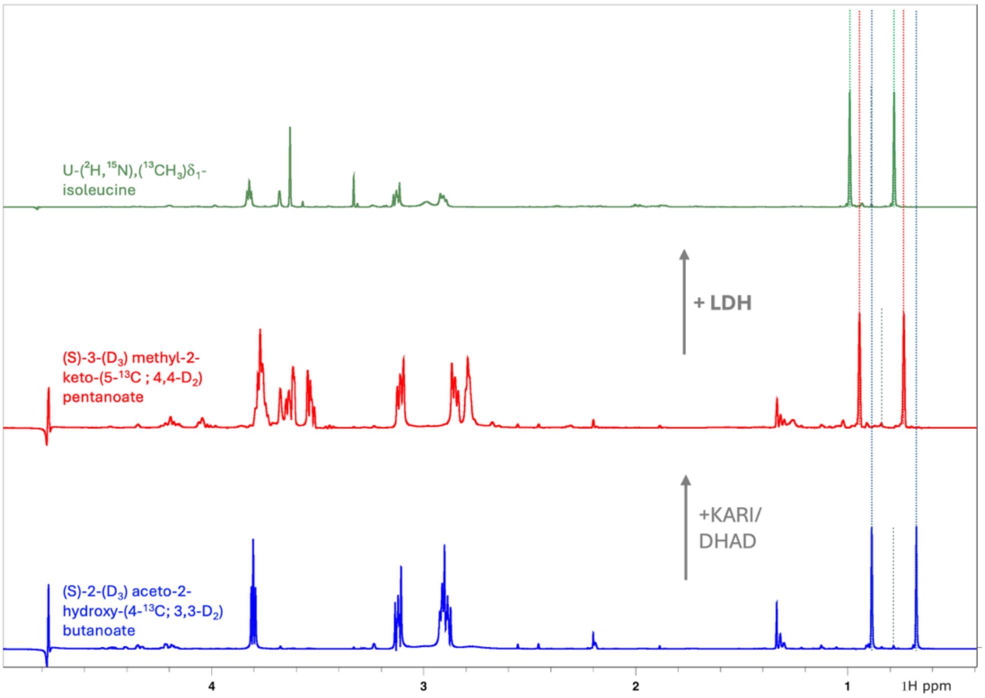


**Figure S2 : Superposition of three 1D ¹H NMR spectra recorded under identical conditions.** The spectra correspond to the precursor (S)-2-($D_3$) aceto-2-hydroxy-(4-$^{13}C$; 3,3-$D_2$) butanoate (blue), the intermediate (S)-3-($D_3$) methyl-2-keto-(5-$^{13}C$ ; 4,4-$D_2$) pentanoate (red) and the final product U-($^2H$,$^{15}N$),($^{13}CH_3$) $\delta_1$-isoleucine (green), illustrating the stepwise transformation from precursor to Isoleucine amino acid.

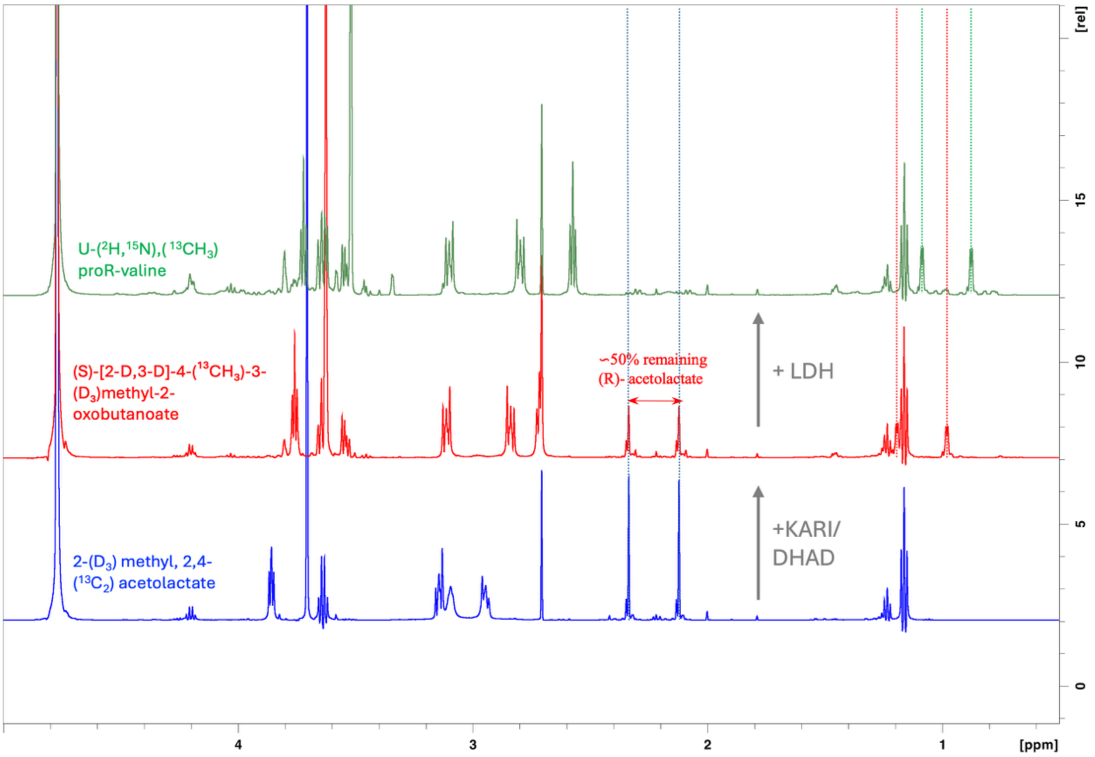


**Figure S3 : Superposition of three 1D ¹H NMR spectra recorded under identical conditions.** The spectra correspond to the precursor 2-($D_3$) methyl, 2,4-($^{13}C_2$) acetolactate (blue), the intermediate (S)-[2-D,3-D]-4-($^{13}CH_3$)-3-($D_3$)methyl-2-oxobutanoate (red) and the final product U-($^2$H,$^{15}$N),($^{13}CH_3$) *pro-R*-valine (green), illustrating the stepwise transformation from precursor to Valine amino acid.

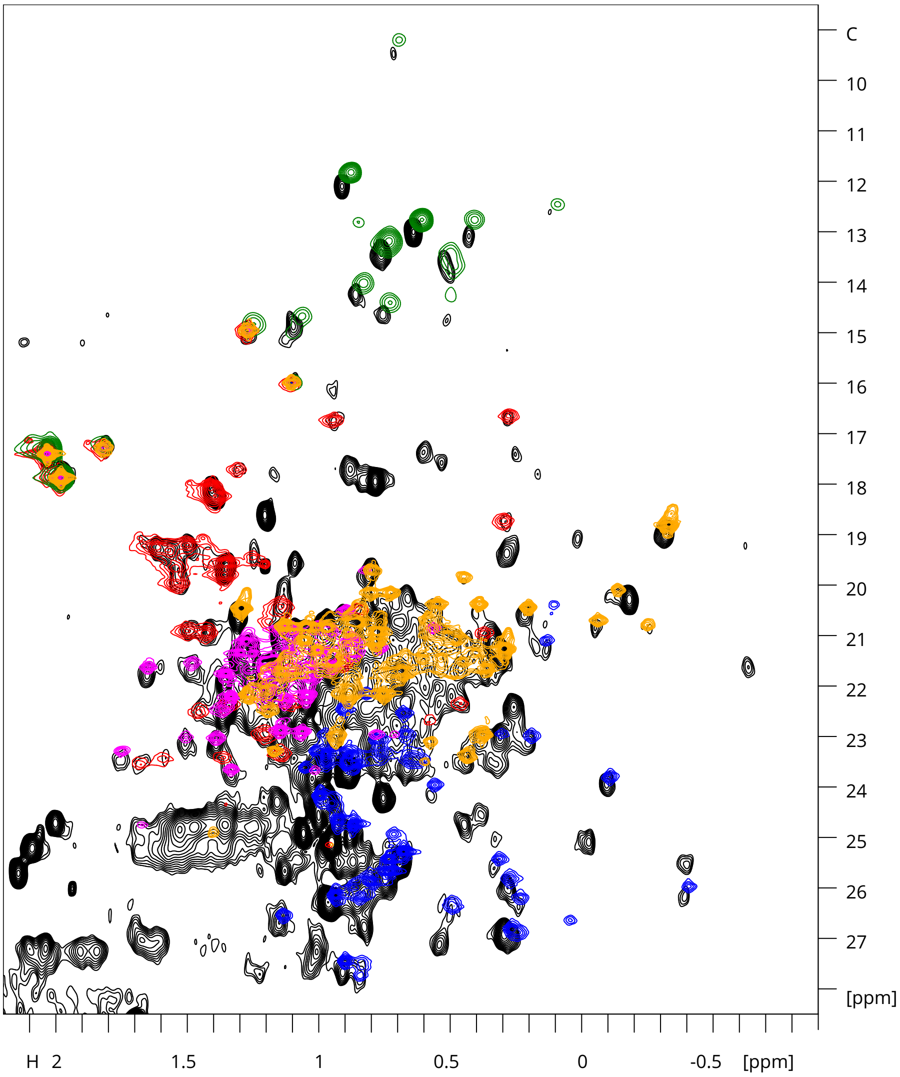


**Figure S4: Overlay of the 2D SOFAST-methyl-TROSY NMR spectra recorded on the methyl-labeled anti-LAMP1 antibody (colored signals: Ileδ1 in green; Ala in red; Thr in pink; Val proR in orange; Leu proS in blue) and the non-labeled anti-LAMP1 antibody expressed in CHO cells (black peaks).** Methionine residues were labeled in all samples, resulting in overlapping peaks across spectra. Experiments were acquired at 35 °C on a spectrometer equipped with a cryogenic probe operating at $^{1}$H frequencies of 950 MHz.

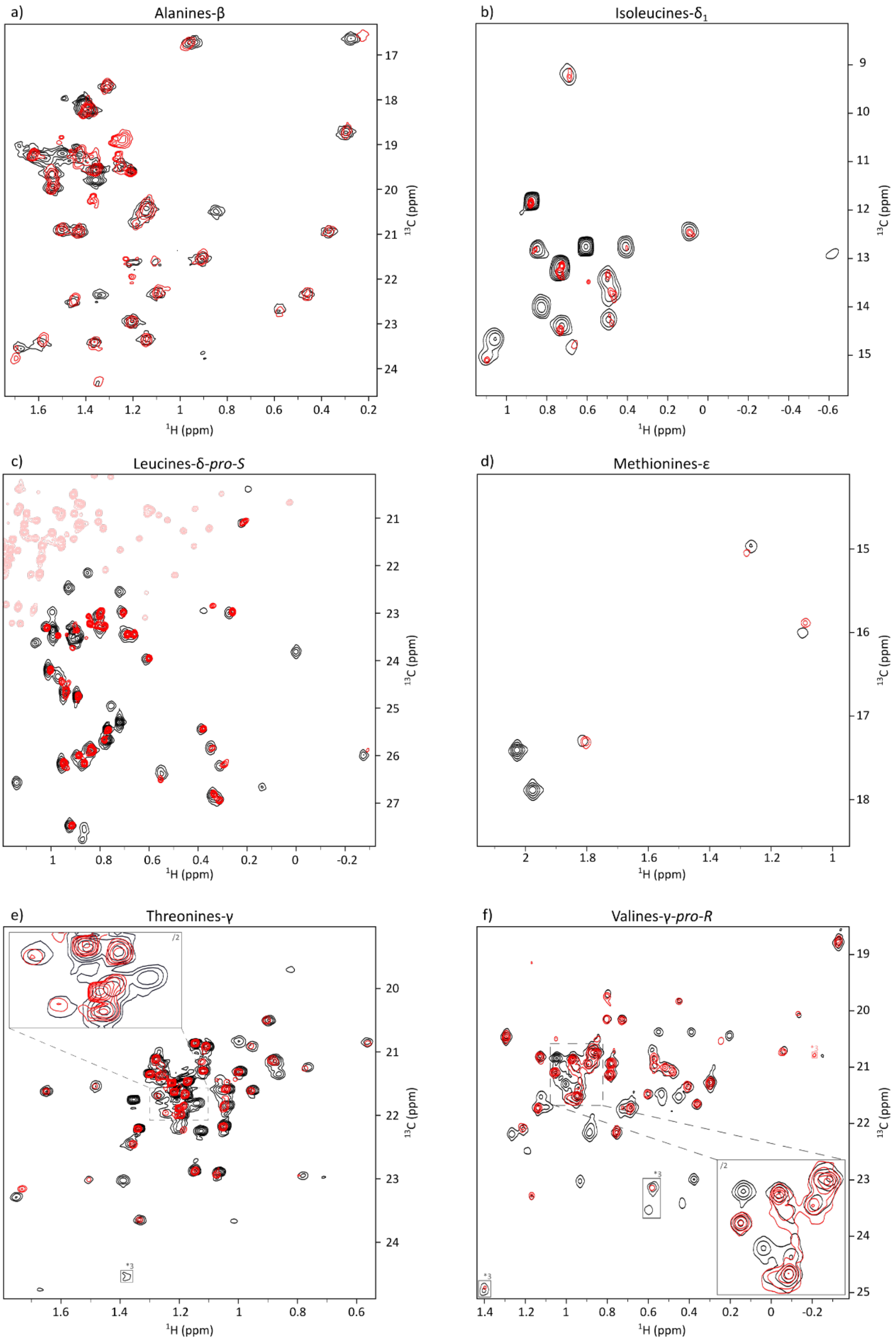


**Figure S5: Overlay of 2D $^{1}$H-$^{13}$C SOFAST-methyl-TROSY spectra of the anti-LAMP1 Fab fragment expressed in a cell-free system (in red) with those of the mAb expressed in CHO cells (in black). a)** mAb labeled on methionines-ε and alanines-β and Fab labeled on alanines-

β, zoom on alanines-β region. **b)** mAb and Fab labeled on methionines-ε and isoleucines-$\delta_1$, zoom on isoleucines-$\delta_1$ region. **c)** mAb labeled on methionines-ε and leucines-δ-*pro-S*, and Fab labeled on alanines-β, isoleucines-$\delta_1$, methionines-ε, leucines-δ-*pro-S*, threonines-γ, and valines-γ-*pro-R*, zoom on leucines-δ region. Signals from the Fab spectrum that do not correspond to leucines are depicted in light red. **d)** mAb and Fab labeled on methionines-ε and valines-γ-*pro-R*, zoom on methionines-ε region. **e)** mAb and Fab labeled on methionines-ε and threonines-γ, zoom on threonines-γ region. **f)** mAb and Fab labeled on methionines-ε and valines-γ-*pro-R*, zoom on valines-γ region. In panels **e** and **f**, contour levels were adjusted for some peaks, which are indicated with grey rectangles.

| $^{13}CH_3$ labeling | Figures | Exp. time | $^1$H frequency | Number of scans | Points in $^{13}$C dimension | Acqu. time $^{13}$C | Acqu. time $^1$H |
|---|---|---|---|---|---|---|---|
| Met, Ala | 3a, 4a | 1h55 | 850 MHz | 48 | 274 | 32 ms | 70 ms |
| Met, Ile-$\delta_1$ | 3b, 4b | 53 min | 600 MHz | 32 | 180 | 30 ms | 50 ms |
| Met, Leu-*pro-S* | 3c, 4c | 3h43 | 950 MHz | 56 | 400 | 33 ms | 70 ms |
| Met, Thr | 3d, 4e | 1h59 | 850 MHz | 48 | 280 | 41 ms | 70 ms |
| Met, Val-*pro-R* | 3e, 4d-f | 3h43 | 950 MHz | 56 | 400 | 33 ms | 70 ms |

**Table S1: Experimental parameters of the 2D [$^1$H,$^{13}$C] SOFAST-methyl-TROSY experiments.** The table lists, for each measurement, the experimental time, the spectrometer $^1$H frequency, the number of scans, the number of points in the indirect ($^{13}$C) dimension, and the acquisition times in both the $^{13}$C and $^1$H dimensions.

| | AAmix1 (mg/L) | Molar % (Culture Medium) | AA sequence fraction (%) | AAmix1_OptimAb (mg/L) |
|---|---|---|---|---|
| Alanine | 93,4 | 4,6 | 5,3 | 107,6 |
| Arginine | 183,4 | 4,6 | 2,4 | 95,7 |
| Asparagine | 260 | 8,6 | 3,9 | 117,9 |
| Aspartic acid | 300 | 9,9 | 4,7 | 142,4 |
| Cysteine | 46 | 1,67 | 2,4 | 66,1 |
| Glutamic acid | 356 | 10,5 | 4,5 | 152,6 |
| Glutamine | 400 | 12 | 4,7 | 156,7 |
| Glycine | 60 | 3,9 | 6,6 | 101,5 |
| Histidine | 50,4 | 1,4 | 2 | 72,0 |
| Isoleucine | 172 | 5,7 | 2,4 | 72,4 |
| Leucine | 92 | 3 | 7,3 | 223,9 |
| Lysine | 169 | 5 | 7,3 | 246,7 |
| Methionine | 208 | 6,1 | 0,8 | 27,3 |
| Phenylalanine | 222 | 5,9 | 3 | 112,9 |
| Proline | 122,4 | 4,59 | 6,8 | 181,3 |
| Serine | 118,4 | 4,94 | 12,4 | 297,2 |
| Threonine | 60 | 2,2 | 8,2 | 223,6 |
| Tryptophan | 24 | 0,5 | 1,8 | 86,4 |
| Tyrosine | 90 | 2,4 | 4,5 | 168,8 |
| Valine | 50,4 | 1,88 | 9,1 | 244,0 |

**Table S2: Amino acid composition adjusted in AAmix1_OptimAb based on antibody sequence**

To obtain the recipe of AAmix1_Optimab, for each amino acid, the target molar fraction was calculated from its percentage in the antibody sequence. For example, alanine represents 5.3% of the total residues in the mAb, while in the original AAmix1 medium it accounted for only 4.6% (93.4 mg/L). The concentration of alanine was therefore adjusted proportionally to match the sequence composition ($93.4 \times 5.3/4.6 = 107.6$ mg/L). This procedure was applied to all 20 amino acids, resulting in the final optimized medium, AAmix1_OptimAb.

| | Ala-β | Ile-$\delta_1$ | Leu-δ-*pro-S* | Met-ε | Thr-γ | Val-γ-*pro-R* | Total |
|---|---|---|---|---|---|---|---|
| **Fc** | 6/7 | 4/4 | 17/18 | 2/2 | 12/16 | 18/23 | 59/70 |
| **Fab** | 28/28 | 12/12 | 29/30 | 3/3 | 16/40 | 37/37 | 125/150 |
| **mAb** | 34/35 | 16/16 | 46/48 | 5/5 | 28/56 | 55/60 | 184/220 |

**Table S3:** Number of assigned resonances on the spectra of the anti-LAMP1 mAb produced in CHO cells and specifically labeled on methyl groups. For each methyl group type, the number of assigned signals is indicated and compared to the number of expected signals.

# Anti-LAMP1 sequence

## Fab - LC

```
 1          10         20         30         40         50         60         70
GDIQMTQSPS SLSASVGDRV TITCKASQDI DRYMAWYQDK PGKAPRLLIH DTSTLQSGVP SRFSGSGSGR DYTLTISNLE
80         90         100        110        120        130        140        150
PEDFATYYCL QYDNLWTFGG GTKVEIKRTV AAPSVFIFPP SDEQLKSGTA SVVCLLNNFY PREAKVQWKV DNALQSGNSQ
160        170        180        190        200        210
ESVTEQDSKD STYSLSSTLT LSKADYEKHK VYACEVTHQG LSSPVTKSFN RGEC
```

## Fab - HC

```
 1          10         20         30         40         50         60         70
GQVQLVQSGA EVKKPGSSVK VSCKASGYIF TNYNIHWVKK SPGQGLEWIG AIYPGNGDAP YSQKFQGKAT LTADTSTSTT
80         90         100        110        120        130        140        150
YMELSSLRSE DTAVYYCVRA NWDVAFAYWG QGTLVTVSSA STKGPSVFPL APSSKSTSGG TAALGCLVKD YFPEPVTVSW
160        170        180        190        200        210        220
NSGALTSGVH TFPAVLQSSG LYSLSSVVTV PSSSLGTQTY ICNVNHKPSN TKVDKKVEPK SC
```

## Fc - HC

```
 221        230        240        250        260        270        280        290
 DKTHTCPPC PAPELLGGPS VFLFPPKPKD TLMISRTPEV TCVVVDVSHE DPEVKFNWYV DGVEVHNAKT KPREEQYNST
300        310        320        330        340        350        360        370
YRVVSVLTVL HQDWLNGKEY KCKVSNKALP APIEKTISKA KGQPREPQVY TLPPSRDELT KNQVSLTCLV KGFYPSDIAV
380        390        400        410        420        430        440
EWESNGQPEN NYKTTPPVLD SDGSFFLYSK LTVDKSRWQQ GNVFSCSVMH EALHNHYTQK SLSLSPG
```
